# The Advantages and Disadvantages of Breakfast Clubs According to Parents, Children, and School Staff in the North East of England, UK

**DOI:** 10.3389/fpubh.2015.00156

**Published:** 2015-06-05

**Authors:** Pamela Louise Graham, Riccardo Russo, Margaret Anne Defeyter

**Affiliations:** ^1^Department of Psychology, Northumbria University, Newcastle upon Tyne, UK; ^2^Department of Psychology, University of Essex, Essex, UK

**Keywords:** school, breakfast, children, families, health

## Abstract

The provision of school breakfast has become increasingly popular in the UK in recent years. However, UK-based studies highlighting the views of parents, children, and school staff on school breakfast clubs are lacking. The current study set out to address this dearth in the literature by investigating the views of these key user and stakeholder groups on breakfast clubs within the North East of England. Fourteen parents, 21 children, and 17 school staff were recruited from four primary schools where breakfast clubs were available on site. Parents and school staff took part in semistructured interviews and children participated in focus groups, through which the advantages and disadvantages of breakfast clubs were discussed. Thematic analysis revealed that breakfast clubs provided children with a settled and enjoyable start to the school day. As well as providing children with a healthy and varied breakfast meal and unique opportunities for social interaction, breakfast clubs were recognized as an integral part of the school system that offered support to parents, particularly those who worked and relied on breakfast clubs as a means of affordable and reliable childcare. The few disadvantages identified related to practical issues such as a lack of adherence to school food standards, breakfast club staff missing class preparation time and concerns that some children were being excluded from participating in breakfast clubs particularly due to costs associated with attendance. The findings are discussed in relation to the School Food Plan, and areas for further investigation are proposed.

## Introduction

Breakfast consumption has been associated with a multitude of health-related benefits, including improved nutrient intake (1), increased moderate-to-vigorous physical activity (2), and improved mood (3). Despite such benefits, breakfast remains the meal that is most regularly skipped (4), which is concerning as breakfast omission has been linked to such problems as increased risk of coronary heart disease (5) and increased body mass index (6).

In an effort to support positive breakfast habits from childhood many schools provide children with an opportunity to consume breakfast on school premises in the company of peers through provision of a breakfast club. Research has shown that breakfast clubs have been somewhat successful in encouraging children to make healthy breakfast choices. For example, studies have shown that children who attend breakfast clubs eat a higher proportion of healthy food items such as cereals and fruits for breakfast than children who do not attend breakfast clubs (7, 8). Though it has also been reported that some breakfast clubs serve foods that are nutritionally poor, thus leading children who attend these clubs to consume more saturated fat and salt than children who do not attend (9).

Although evidence surrounding the effectiveness of breakfast clubs is mixed, the provision of breakfast in UK schools was recently advocated within the School Food Plan [SFP (10)]. The SFP sets out actions to be completed in schools with the support of multiple organizations, including Government and public health representatives. The SFP aims overall to improve the provision and uptake of school food throughout the school day while educating children, parents, and school staff on the importance of good nutrition. The SFP has received support from the Secretary of State for Education and substantial financial investment from UK Government to support the implementation of the agreed actions. In terms of breakfast provision, the SFP specifies that funding will be allocated to support the setup of sustainable breakfast clubs in England”s poorest schools during the next 2 years, even though at present very few UK-based studies have investigated the effectiveness of breakfast clubs. Moreover, the views of key stakeholders and users who are directly affected by policy changes are underrepresented within the research literature. Given that the success of the SFP relies on support from school staff, children, and parents, input from these groups on school food programs is likely to play a crucial role in school food development into the future. The aim of the current research is to address the paucity of research in this area. Specifically, focus groups with school children and semistructured interviews with parents and school staff will be employed to better understand what users and key stakeholders think are the advantages and disadvantages of school breakfast clubs.

## Method

### Approach

The views of parents and school staff were obtained through semistructured interviews. Interviews were considered an optimal method of data collection for adults as previous studies have shown that questionnaire response rates are often low, particularly from low-income parents (11). In comparison, interviews are thought to be more rewarding for participants as they involve a social exchange, thus people are more likely to want to participate (12). Interviews also allowed adults to express their views about sensitive topics such as school procedures and parental care practices without the risk of being reprimanded by fellow participants. Children took part in small focus groups with two or three peers of similar age. Small focus groups have been advocated as a method of collecting data from children as they are similar to small group discussions that children are involved in during their normal school day (13). Such familiarity is likely to ensure that children feel at ease taking part in research.

### Participants

The current investigation adopted a purposive method of sampling. Purposive sampling is a non-probability sampling technique that involves the recruitment of participants on the basis of a particular characteristic of interest (14). While purposefully selecting participants on the basis of certain criteria might introduce some bias, it has been argued that “The inherent bias of the method contributes to its efficacy, and the method stays robust even when tested against random probability sampling. Choosing the purposive sample is fundamental to the quality of the data gathered” (15, p. 147).

In the current study, 14 parents, 17 school staff, and 21 children were purposively sampled as they were all familiar with their school breakfast club. All participating parents had at least one child attending breakfast club and were responsible for between one and six dependent children within their household at the time the interviews took place. Two parents were in full-time employment, six parents were in part-time employment, one was self-employed, two were in voluntary roles, and three were unemployed. Four of the parents interviewed were also volunteers in their children’s breakfast clubs, helping with the serving of breakfast and clearing away, and one parent attended breakfast club with her children. All staff had knowledge of their school breakfast club and were, therefore, able to express opinions on it. Three teaching assistants, one lunch time supervisor, and one social inclusion officer were involved in the day to day running of breakfast clubs at the time the interviews took place. The head teacher and trainee teacher had been involved in the initial setup of breakfast clubs within their schools. The remaining staff members (six class teachers and four teaching assistants), who did not work in breakfast clubs and were not involved in their setup, all worked directly with children who attended breakfast clubs within their classes on a daily basis. Participating children were aged between 4 years 8 months and 11 years 1 month (mean age =7:9) and breakfast club staff confirmed that all children had frequently attended their school breakfast club during the 3 weeks prior to taking part in the focus groups.

Participants were recruited from four primary schools based in predominantly White British, low-income areas of the North East of England. The characteristics of the participating schools, their breakfast clubs, and the areas that the schools resided in are presented in Table 1. Table 1 shows that in Schools 1 and 4, a smaller proportion of children were entitled to free school meals than in Schools 2 and 3. As free school meal entitlement is often used as an indicator of socioeconomic status (16), these figures could suggest that Schools 2 and 3 had higher levels of deprivation than Schools 1 and 4. However, the unequal numbers of participants recruited from each school (see Table 1) meant that it was not possible to make comparisons between schools on the basis of potential socioeconomic differences. The breakfast clubs, which were the focus of the study, were quite similar across schools with only one school offering breakfast to children free of charge. All the breakfast clubs were well established, having been in place for between 6 and 17 years. The clubs were all available Monday–Friday during term time only. Any differences in the views of participants possibly relating to the cost of breakfast club attendance are highlighted within the results section.

**Table 1:** **Characteristics of participating schools, school areas, and breakfast clubs**.

Schools	School demographics			School local area demographics		Breakfast club characteristics			Recruitment
	
	Pupils on roll (*N*)	School type	Pupils entitled to free school meals (%)	All people of working age claiming a key benefit (%)	White British (%)	Cost and availability	Activities	Breakfast	Number of participants recruited from each school
1	262	Voluntary aided	21	22	97.9	£2.00 per day 8:00 a.m.–8:50 a.m.	Ball games, skipping, toys, drawing	Cereal, toast, pancakes, juice, fruit	Parents = 1 Children = 2 Staff = 8
2	287	Community	41.4	34	97.9	£1.50 per day 8:00 a.m.–8:30 a.m.	Table top activities, reading, toys	Cereal, toast, yogurt, fruit, juice	Parents = 5 Children = 6 Staff = 6
3	228	Voluntary aided	58.7	37	94.9	Free (externally funded) 8:00 a.m.–8:55 a.m.	Arts and crafts, board games, reading	Cereal, toast, yogurt, juice	Parents = 6 Children = 10 Staff = 3
4	285	Community	18.2	19	98.0	£2.50 per day for 7:30 a.m. arrival £2.00 per day for 8:15 a.m. arrival 7:30 a.m.–8:55 a.m.	Drawing and coloring, construction, television, books, board games, physical activity games	Cereal, toast, yogurt, fruit, juice	Parents = 2 Children = 3 Staff = 0

### Materials

Three separate schedules of open-ended questions were devised to guide interview and focus group discussions. Although the questions differed slightly between schedules to ensure that they were suitably worded for each participant group, the questions focused on the following topics: What happens at breakfast club and why do children attend? What difference does breakfast club make to children, parents, and schools? Are there any issues associated with the utilization of breakfast clubs? What are the positive aspects of breakfast clubs? What could be done to improve breakfast clubs? What would the impact be if breakfast clubs were to close?

### Procedure

Following ethical approval from the University Ethics Committee, opt in consent was obtained from all adult participants prior to commencement of interviews. Parental consent was obtained for all children prior to the focus group sessions, and children provided verbal assent immediately prior to taking part in the focus groups. A semistructured format was utilized throughout to allow the interviewer to gain further insight into points of interest and to seek clarification from participants when necessary. All interviews and focus groups were conducted on school premises by an independent researcher and were audio recorded and transcribed verbatim for subsequent thematic analysis.

### Analysis

#### Sample Size and Saturation

While there is much debate surrounding the issue of what should be considered an adequate sample size in qualitative research (17), it has been suggested that interviews should continue until a saturation point is reached. Saturation refers to the point at which no new ideas are identifiable within the data set (18). It has been argued that qualitative researchers should not continue to collect data beyond the point of saturation simply to increase the number of participants in the sample as the additional participants do not add anything to the analysis (19). In a paper by Guest et al. (20) where the concept of saturation was investigated, the researchers found that a sample of 12 participants was an adequate number to reach saturation. Similarly, Francis et al. (21) proposed a “10 + 3” method, which involves analyzing the data of 10 participants then continuing to interview until three further consecutive interviews reveal no new themes. In the current study, the analytical process was carried out in parallel with interviews and focus groups to allow the research team to determine when a saturation point was reached. As the current investigation did not set out to make comparisons between schools but was instead interested in the views of key groups (i.e., parents, children, and school staff), data were collected until the research team were confident that a saturation of themes within each key group had been reached across schools.

#### Thematic Analysis

Data were coded and analyzed according to published guidelines on thematic analysis (22). Each individual recording was listened to in its entirety to ensure familiarization before being orthographically transcribed to capture the content of discussions with parents, school staff, and children. Each transcript was read numerous times then pertinent points thought to refer to any advantages and disadvantages of breakfast clubs were highlighted. NVivo version 10.0 software was used for the storage of highlighted quotes and further categorization. The highlighted quotes were given labels to summarize the topics they referred to and similar topics were grouped together. Main themes were developed from the topic groups, and appropriate theme headings were generated to summarize the data being presented. This inductive approach to thematic analysis was adopted as there is currently no published theoretical framework on breakfast clubs on which the current analysis could have been based. It is further argued that “The keyness of a theme is not necessarily dependent on quantifiable measures – but in terms of whether it captures something important in relation to the overall research question” (22, p. 10). Moreover, the point of qualitative research is to focus on the insights of the participants relating to a particular phenomenon rather than the number of participants that share a certain view point (23). For this reason, the proportion of participants who shared similar views are not reported, though the main themes were evident across all schools. To confirm the accuracy of the coded transcripts, a second coder analyzed around 10% of the data. Agreement between the first and second coder was found to be very good (Cohen’s kappa =0.905; *p* < 0.001).

## Findings

Parents, children, and staff spoke favorably about breakfast clubs. They identified a multitude of advantages associated with school breakfast provision that extend beyond nutritional enhancements alone. Some minor areas for improvement were also proposed. An overall summary of the themes identified by parents, children, and staff is presented in Table 2 followed by a detailed analysis of the themes with example quotes provided to support each theme. Individual schools are not identified alongside quotes to ensure that participant anonymity is upheld. According to the British Psychological Society’s (BPS) Code of Human Research Ethics (24), “Participants in psychological research have a right to expect that information they provide will be treated confidentially and, if published, will not be identifiable as theirs” (p. 22). If the authors were to identify which school the participants were taken from, they could potentially breach these ethical guidelines. As there was, for example, only one head teacher and one social inclusion officer involved in the study and these roles are identified alongside their quotes, if the schools were also specified it may be possible for a reader to combine this information to identify the individual with whom the quote is associated. It is especially important that the identities of our participants are protected as some raised issues with breakfast club practices that brought into question the decisions made by school senior management teams. The research team chose to conduct individual interviews rather than focus groups with adults to allow participants to express their views without the risk of causing issues with those they might disagree with. The research team therefore chose to maintain the highest level of participant anonymity possible.

**Table 2:** **Summary of themes outlining the advantages and disadvantages of breakfast clubs**.

Themes	Main points made by parents	Main points made by staff	Main points made by children
Breakfast meal	Good variety of healthy foods Children more willing to have breakfast Children can try new foods without parents having to buy and risk wasting larger quantities Some concerns regarding sugar added to cereals Greater variety of cereals wanted	Breakfast provided for those who would otherwise skip Cost of attendance excluded some children Children more willing to eat with peers Greater variety at breakfast club than at home Generally healthy but some unhealthy items served Some disagreement over the necessity to adhere to school food standards	Good variety of foods available Breakfast provided for those who would otherwise skip Children can try new foods that they would not have at home
Social opportunities	Time for informal interaction and relationship developmentx Access to a broader peer group Reduces social limitations	Time for informal interaction and relationship development Access to broader peer group Reduces social limitations	Time for informal interaction and relationship development Access to broader peer group
Means of support	Reliable form of childcare especially useful for parents who work/study and those without extended familial support Peace of mind that children are safe before school	Reliable form of childcare especially useful for parents who work/study and those without extended familial support Peace of mind that children are safe before school Accessible means of communication between parents, children, and school Some barriers to fully inclusive provision	
Positive start	Calmer household in the mornings Enjoyable start to the day for children Helps punctuality Children more prepared for school	Calmer, more enjoyable start to the day for children Helps attendance and punctuality Concerns surrounding uncertain financial support for intervention Children more prepared for school Extended school day for children	Enjoyable start to the day Better than alternative optionsb Helps punctuality Children more prepared for the school day Some reported tiredness but not attributable to breakfast club *per se*
School practicalities		Integral part of school Staffing needs consideration Designated breakfast club space would be advantageous	Array of enjoyable activities for children Updates needed to equipment Children would like more peers to attend

### What are the advantages and disadvantages of breakfast clubs according to parents?

Four key themes encompassing the advantages and disadvantages of breakfast clubs were identified through interviews with parents. The themes pertained to the breakfast meal, social opportunities, parental support, and positive start to the day. Each theme is subsequently discussed in detail.

#### Theme 1: Breakfast Meal

The provision of a breakfast meal was generally recognized as an advantage of breakfast clubs. Parents talked positively about the variety of breakfast items made available to their children:

There’s always plenty for them to choose from to eat, there’s plenty for them to choose from to drink (Mother of one child; self-employed)

Parents also considered the food and drink items available at breakfast clubs to be healthy:

In the main he gets I’d say a nutritious breakfast (Mother of two children; voluntary work)

Though there were caveats to this as a minority of parents (n = 3), when asked what they would change about breakfast club, offered suggestions relating to foods. Two of the parents in question mentioned that the provision of a greater variety of cereals would improve breakfast club:

I think more of a variety of like cereal; I think the kids get bored of the cereal (Mother of two children; unemployed)

Also, although the addition of sugar to cereals was allowed under the food-based standards (25) in place at the time data were collected, two parents expressed concerns that children were able to add sugar to cereals in breakfast clubs and felt this should be limited:

Nothing serious or major it’s just she asks for a lot of sugar on her cereal and I wouldn’t normally give her that much sugar (Mother of one child; voluntary work)

However, parents’ views on the provision of breakfast within breakfast club were predominantly positive and this provision was associated with more favorable breakfast habits in children. Some parents faced difficulties with trying to persuade their children to eat breakfast at home in the mornings before school:

It would be a nightmare ’cause the kids wouldn’t have breakfast so they’d be coming to school hyper ’cause all they’d want is biscuits (Mother of six children; unemployed)

However, parents suggested that their children were more willing to eat breakfast at breakfast club than at home:

I know for definite he eats. I know sometimes at home he can be a bit take it or leave it, I know he’s definitely getting it here (Mother of one child; employed part time)

Moreover, children were more likely to try new foods at breakfast club that they would be reluctant to try at home:

She won’t have apple juice at home but she’ll have it here you know so she does actually try different things here (Mother of one child; self-employed)

In addition, breakfast clubs allowed children to try new foods and drinks without parents having to buy and risk wasting breakfast items in bulk:

At home I’d have to buy a full box of it and if she doesn’t like it it’s a waste so I think a bit of variety gives them a bit more choice instead of having to buy the cereal first and then bin it if she doesn’t like it (Mother of five children; employed part time)

Children having opportunities to taste new foods in breakfast clubs was also suggested to have a subsequent impact on family shopping habits:

I said to them before, pick your cereal, they never wanted to try Cheerios, they come here, they try Cheerios, now they love Cheerios so we buy them at home (Mother of four children; employed part time)

Parents typically viewed the provision of breakfast at school to be favorable as it offered their children an opportunity to consume a healthy and varied breakfast meal and in some cases it encouraged children to eat breakfast when they would otherwise skip it at home. However, there were some concerns raised surrounding the lack of variety of cereals available in breakfast club and the addition of sugar to cereals by staff.

#### Theme 2: Social Opportunities

The social opportunities afforded to children through breakfast clubs were highly valued among parents. They talked amiably about the way in which breakfast clubs allowed their children to spend time interacting informally with their peers before the start of the school day:

They can relax and chill with their friends before the school day starts to me that’s one of the biggest advantages (Mother of five children; employed part time)

The time that children spent with one another within breakfast clubs was further suggested to be conducive to children’s relationship development:

They also make a lot more relationships in breakfast club as well they’ve made a lot more friends (Mother of two children; employed full time)

Furthermore, all the breakfast clubs involved in the current study catered to children of various ages across the school, thus allowing children to spend time with peers that they might otherwise be unable to spend time with:

He’s sort of made friends with urm different age groups as well within there and he talks about other children now as opposed to some of the class children that he’s obviously- he spends a lot of time with (Mother of five children; unemployed)

Finally, it was evident that some children faced limitations in their abilities to socialize with peers that could be attributed to developmental factors such as autism or environmental factors such as limited play space at home. Breakfast clubs were recognized as a beneficial environment for helping children to overcome such social limitations as they provided space for children to engage informally with peers and to learn from the social abilities of others:

My daughter has autism so she actually gains from a social side of being in breakfast club in a formal way and getting on with other children and having a bit more free time, free space to do different things just watching what other children do (Mother of five children; employed part time)

The social opportunities discussed by parents were wholly positive. Parents viewed the informal time in breakfast club as greatly advantageous as it allowed their children to form friendships across a diverse social group and helped some children to overcome barriers to social interaction.

#### Theme 3: Means of Support

It was evident that breakfast clubs were a valuable means of support for parents, providing a reliable form of childcare that was especially useful for parents engaged in study or employment:

I think it’s really good for parents, especially obviously parents that are working (Mother of one child; employed full time)

The provision of breakfast clubs allowed parents more flexibility in terms of their availability for work and many parents stressed that without breakfast clubs they would be unable to maintain their current employment role:

If it wasn’t for the breakfast club I wouldn’t be able to go to work “cause I start work at quarter past eight so it’s a case of dropping them off here at eight and then making my way straight to- straight to work so really it’s essential for me to go to work really (Mother of three children; employed part time)

Further to this, it was clear that parents often had to rely on breakfast clubs to support their childcare needs as they did not have a wider familial support network available. This was typically due to family members living considerable distances away from one another and family members being unavailable due to their own work commitments:

His dad works away so it’s- I’ve got kind of on a morning it’s just me and [child] so I’ve got no one to rely on on a morning to get him to school (Mother of one child; employed full time)

In addition, parents placed great emphasis on the safe environment that breakfast clubs provided for their children before the start of the school day. Knowing that they were leaving their children in a secure environment on the school premises where they would be properly supervised and given breakfast before the start of the school day provided parents with peace of mind:

Just knowing that he’s actually safe as I’m on my way to work and stuff is reassurance for me (Mother of one child; employed full time)

Overall, breakfast club provision was invaluable to working parents. It allowed them to leave their children on the school premises before the start of the formal school day safe in the knowledge that their children were being adequately cared for. This was especially helpful for parents who felt they lacked other means of support and without breakfast club would be forced to change their work pattern.

#### Theme 4: Positive Start to the School Day

The final theme discussed by parents related to the positive start to the school day that breakfast clubs provided for their families, which began within the family home in the mornings. Parents talked about the way in which the provision of breakfast in school led to there being less of a rush in their household in the mornings:

We can all be a little bit more relaxed in the morning and at least I know they’re going to get something rather than trying to rush them into getting some breakfast (Mother of three children; employed part time)

In particular, parents felt that there was less conflict within their homes before breakfast club. Parents found that they did not encounter issues with trying to persuade their children to eat breakfast and children were less likely to fight with siblings; they simply got up and dressed and left the house in a more efficient manner for breakfast club:

If they’re at home all they do is fight and argue but they’re alright in school, they’re calm (Mother of six children; unemployed)

Furthermore, parents were satisfied that breakfast clubs provided their children with an enjoyable start to the day. No parents suggested that their children had any reluctance to attend breakfast clubs at any time:

He’s gone no problem, he absolutely loves it (Mother of one child; employed part time)

For some, their child’s enjoyment of breakfast club led to them attending more days than was absolutely necessary:

Even if I’m on holiday at work she still goes Wednesday, Thursday, Friday “cause she still enjoys it so she still comes (Mother of one child; employed part time)

The positive start to the day provided by breakfast club in the family home also had important school related benefits. For a number of families, the provision of breakfast club helped them to get their children to school on time. On days when children were expecting to go to breakfast club, they were more co-operative with parental directives to get up and ready efficiently:

If I wake her up in the morning and say we’re at breakfast club this morning she will get up easier than she gets up if it’s just normal school (Mother of one child; voluntary work)

Additionally, breakfast clubs were noted as being particularly beneficial for those families who had experienced severe difficulties with attendance and punctuality in the past:

If I don’t come to breakfast club I wouldn’t get them to school on time (Mother of six children; unemployed)

Finally, parents suggested that breakfast clubs ease children into the school day and leads to them being more alert and ready to learn at the start of the formal school day:

I think it does get them started ready for the day, like I say by the time the school starts they’ve been up two hours, some kids you see them walking to school and they’re asleep you know so it just helps them to start the day (Mother of three children; employed part time)

In sum, parents suggested that that their family homes could be challenging in the absence of breakfast club provision. Breakfast club was thought to provide a calmer start to the day with less of a rush, fewer family conflicts and an enjoyable time for children.

### What are the advantages and disadvantages of breakfast clubs according to school staff?

Staff views on breakfast clubs were similar to those of parents with advantages and disadvantages for children, families, and the school being highlighted.

#### Theme 1: Breakfast Meal

First, staff suggested that they were aware of children in their school who tended to skip breakfast at home:

From the area what we live in, there are lots of children that don’t get breakfast (Trainee Teacher)

Breakfast provided through breakfast club was thought to alleviate this issue. In one club, where breakfast was available to whole families, staff reported that parents would take full advantage of this:

We have some parents who actually can’t afford to give their children breakfast so like a full family will come for breakfast (Teaching Assistant and Breakfast Club Staff)

However, it was also argued that where breakfast club attendance incurred a charge, some children who could benefit from the availability of breakfast at school were excluded due to costs:

Those who don’t get breakfast before they come to school are very often children who would benefit more from it, however there’s the cost involved so that’s a reason for people not sending children (Class Teacher)

This was evident as despite breakfast club being available, some children arrived at school hungry and staff felt it necessary to provide them with breakfast at the start of the school day:

We’ve had two girls particularly who’ve came and said I haven’t had breakfast this morning. [Breakfast club staff] and I do go and look for something to try and give them (Class Teacher)

Though it should also be pointed out that there was a perception among staff that some children skipped breakfast as they chose not to have it at home. In such cases, breakfast club was reported to provide an environment where children would be more willing to eat breakfast:

Children will eat better ’cause there’s other children about. I know some parents have sent children because of that (Class Teacher)

In terms of the foods served, breakfast clubs were thought to allow children to try a greater variety of foods than they have available to them at home:

I think encouraging children to eat different things and try different things it maybe gives them that opportunity to eat things that they wouldn’t eat at home (Class Teacher)

In some cases, the breakfast meal at breakfast club was thought to be healthier than breakfast provided at home:

They’re not coming to school with a packet of crisps or a bar of chocolate or a fizzy drink. At least you know what they’re getting (Social Inclusion Officer and Breakfast Club Staff)

However, some staff pointed out that unhealthy breakfast items were being made available to children in breakfast club:

There’s a variety of breakfasts, I know there’s only a certain amount of what would be classed as unhealthy stuff they’re allowed each week (Class Teacher)

One member of staff expressed particular concern about this issue as she felt that some breakfast options should be changed but a lack of support from senior management meant that she felt powerless to be able to enforce any changes:

If it’s not stamped on by people who are you know higher management in this school than me I’m not prepared to get into arguments with members of staff over it so that’s kind of where I would back track (Teaching Assistant)

This issue was further highlighted and not unique to one particular school as one member of staff supported the serving of healthy and unhealthy breakfast options in order to educate children about nutritional balance:

You’ve got to show them these are your options available, if you just ate that there’s too much sugar and fat but if you have some of that and some of that and some of that that’s a balanced diet (Head Teacher)

Overall, the theme of “Breakfast Meal” shows that breakfast served through breakfast club has the potential to ensure that children who skip breakfast at home, whether through choice or necessity, have the option to have a meal before the start of the school day. Moreover, in some cases, breakfast club might reduce the likelihood of children consuming foods of low nutritional value for breakfast (e.g., sweets and crisps). However, it was apparent that there are issues with some of the items served for breakfast in breakfast clubs as school food guidelines, which are in place to govern the content of all school food menus, are not being followed and school senior management teams are willing to allow such guidelines to be contravened. Furthermore, while breakfast clubs were suggested to support some children in obtaining breakfast when it is skipped at home, concerns were raised that the cost of breakfast club attendance resulted in some children being excluded from accessing breakfast within breakfast club.

#### Theme 2: Social Opportunities

Staff recognized great value in the social opportunities afforded to children and breakfast club staff through involvement in breakfast club. The time spent in breakfast club was believed to provide time for children to engage in informal interaction with peers and staff before the start of the school day:

You can have a little bit of chatter, you can chat- last night, did you watch the football last night? Load of rubbish weren’t they? (Teaching Assistant and Breakfast Club Staff)

This informal time was thought to be conducive to the development of positive relationships not only between peers but also between children and breakfast club staff:

They have a very good relationship with [Breakfast Club Staff]; the children speak very fondly of her (Class Teacher)

Moreover, the mixed age groups that participated in breakfast clubs allowed children to socialize with peers that they might not otherwise have the opportunity to spend time with:

These are like from the eldest of our children down to the smallest of our children and they’re mixing so it’s giving them a different experience than what they would have just in the playground with other kids (Social Inclusion Officer and Breakfast Club Staff)

It was also suggested that without breakfast clubs, some children would be more limited in their opportunities to interact with others before school:

Sometimes if you’re a parent, you’ve only got one child, they just sit and eat their breakfast in silence “cause you’re too busy getting ready for work or whatever (Class Teacher)

Staff views on the social opportunities that breakfast clubs provide mirror suggestions put forward by parents proposing that the social aspect of breakfast club attendance is advantageous. Time spent in breakfast clubs allows children to interact with a more diverse group than they would typically throughout the school day, which is thought to encourage the development of relationships and reduce social isolation that might feature in some households in the mornings.

#### Theme 3: Means of Support

Staff highlighted numerous ways in which breakfast clubs provide various forms of support that have wide reaching implications for families and the school community. The support that breakfast clubs offered working parents received particular attention throughout discussions with staff. It was suggested that breakfast clubs provided a valuable form of affordable childcare that allowed many parents to work:

When you work, if you- you need childcare- child support so if we’re offering ur- ur- an alternative to nursery then it’s fair, it’s fair you know otherwise a parent might not be able to work and the breakfast clubs a lot cheaper than a child minder, a lot cheaper (Class Teacher)

It was further suggested that the availability of childcare before school was crucial for some families as before school childcare is much more limited than after school care:

It’s easier I think to make arrangements after school if you need to for meetings and things than it is before school, there are not many- whereas they might want to go- to go to grandma’s or their friends house or something after school, people generally don’t want other people in their houses in the morning (Class Teacher)

Additionally, breakfast clubs were thought to support families by offering a flexible form of childcare that is easily accessible at short notice:

If mam’s got an appointment at hospital we can just say to her, even if it’s just a one off, fill a form in and you can come to- we can give you breakfast club for that day (Teaching Assistant and Breakfast Club Staff)

Staff believed that the availability of breakfast club provided parents with peace of mind by offering a safe environment in which to leave their children before the start of the formal school day:

It’s knowing that there’s somebody there to look after them and that they’re gonna be well looked after and that they’re not worrying about getting them to school and that they’re safe (Trainee Teacher)

Furthermore, staff felt that breakfast clubs supported parents by providing a means of communicating with school. This was particularly useful for parents who lacked confidence in approaching school staff in a more formal manner:

It is hard for our parents who haven’t got a lot of social skills to come into the main office to ask something where it’s easy to just ask us sometimes (Teaching Assistant and Breakfast Club Staff)

Staff also believed that breakfast club offered children an important outlet for communication. The availability of staff within breakfast club meant that children had someone to talk to if they needed help and issues could be dealt with before children entered class:

She builds up that rapport with them and they have somebody who they can confide in (Class Teacher)

However, schools were not always able to provide an adequate level of support to be able to offer fully inclusive provision. One head teacher suggested that they were unable to allow children requiring individual support to attend breakfast club as this was not a financially viable option:

There was a little boy who had one to one support in school and his mam wanted him to come but we couldn’t take him because the two pound a day that wouldn’t even cover the cost of staffing (Head Teacher)

Thus, in some cases, children were being excluded from attending breakfast club due to cost implications.

The views of staff generally supported those of parents to suggest that breakfast club provision is highly valued as a means of reliable and affordable childcare. Breakfast club also gave parents and children a useful mechanism for communicating with school before the start of the school day. Unfortunately, the costs associated with the staffing of breakfast club meant that schools could not offer a fully inclusive service for all families.

#### Theme 4: Positive Start to the School Day

Staff talked about multiple factors relating to breakfast clubs that could help provide children and their families with a more positive start to the school day. First, staff believed that breakfast clubs provide children with a calmer and more enjoyable start to the school day than they might encounter at home:

It’s much more calm atmosphere where they can sit, chat to friends, eat breakfast, without the stress of parents pushing them and getting them moving to leave on time (Class Teacher)

This was thought to be the case particularly for children whose families face difficulties:

There’s probably some children who might be better off in breakfast club than what they see at home (Teaching Assistant and Breakfast Club Staff)

In addition, breakfast clubs were thought to prepare children for the school day by providing routine:

It kind of sets them up for the day really, they’re into school mode and it becomes part of their little routine and they’re a little bit more alert I think by the time they’re coming to school (Head Teacher)

For some children, the breakfast club routine supported them to arrive punctually so they did not miss the start of the school day:

Without it we’d have a lot more children who would be absent, wouldn’t get in on time, who’d be late (Teaching Assistant and Breakfast Club Staff)

However, in one school where breakfast club was being used as an intervention to address issues of lateness with some children, there were concerns that when temporary financial support ended, these children would return to being late:

I think when social care stops paying their money they might slip back into the routine (Teaching Assistant and Breakfast Club Staff)

An additional caveat to the potential positive start to the day provided by breakfast clubs is that breakfast club attendance extends the duration of the school day for children. Some staff therefore expressed concerns that children attending breakfast clubs spend a long time on the school premises, which could consequently impede on family time:

We’ve had some children who go to breakfast club and they go to after school club so their parents drop them off at eight o’clock in the morning and they don’t see anybody else from their family “til half past five (Class Teacher)

Overall, staff proposed that breakfast club attendance could be advantageous to the start of the school day as it provides children with a calm environment, which might be better than the home setting for some children. Breakfast club also provides routine and structure, which was thought to support school attendance and punctuality and prepare children for the school day. Though, the potential for breakfast clubs to act as an intervention to support punctuality without long-term financial investment was questioned. Finally, staff had some concerns that breakfast clubs extended the school day and reduced the amount of time that children spent with their families.

#### Theme 5: School Practicalities

Staff viewed breakfast clubs as an integral part of schools that played a key role in the external image of those schools:

It gives the school a good spec in terms of wraparound care that type of thing so in terms of OFSTED and the public image of the school and the facilities we can provide that’s a good thing (Class Teacher)

However, there were suggestions that more consideration needs to be given to the staffing of breakfast clubs and the setting in which they take place. In terms of staffing, it was suggested that breakfast clubs could benefit from having more staff available to allow more children to attend though in some schools breakfast clubs were understaffed with the number of children already involved:

I know they’re rushed and they don’t have enough time… sometimes there’s only one of them and if it’s a full breakfast club (Teaching Assistant)

Moreover, there were concerns that breakfast club responsibilities took some staff away from duties that they were required to do as part of their main role within school. This meant that breakfast club staff had to arrive at school extra early to fulfill all their duties or another member of staff had to take on additional tasks while breakfast club staff were in breakfast club:

Where other TA’s are in at half eight and they’re there for twenty minutes doing photocopying and getting ready for the day, I do all of it myself because [Breakfast Club Staff] can’t (Class Teacher)

As well as relying on existing school staff, breakfast clubs tended to run in areas that were utilized predominantly for other activities so breakfast club was a secondary activity within these settings. Staff suggested that having spaces and facilities designated specifically for children attending breakfast clubs would be advantageous:

In an ideal world I think it would be more child friendly in terms of furnishings and look and feel and welcoming atmosphere rather than quite an austere old persons building (Class Teacher)

It was evident through discussions with staff that breakfast clubs were valued within schools but the theme of “School Practicalities” demonstrates that more consideration needs to be given to breakfast club staffing and facilities to ensure they are adequate and do not negatively impact other aspects of school life.

### What are the advantages and disadvantages of breakfast clubs according to children?

The views of children generally supported those of parents and school staff with discussions surrounding the provision of breakfast, social time, and support.

#### Theme 1: Breakfast Meal

Children enjoyed a variety of breakfast items made available to them through breakfast club:

I like going to breakfast club because you can have anything, you can have cereal and toast (Girl, aged 7 years)

The provision of school breakfast allowed children to try foods that they would not have at home:

You get Frubes [yogurt] there, I wouldn’t get Frubes for breakfast [at home] (Girl, aged 10 years)

It also prevented some children from skipping breakfast. As suggested previously by parents and staff, children skipped breakfast for different reasons; some chose to skip breakfast:

Well sometimes I don’t really have breakfast… “cause like I can’t be really be bothered (Girl, aged 7 years)

While other children skipped breakfast due to a lack of food in the family home:

We haven’t got no food have we [brother]? (Boy, aged 5 years)

Overall, it was evident that children valued the breakfast provided through breakfast club. It offered them a greater variety of breakfast options and more importantly prevented some children from skipping breakfast before the start of the school day.

#### Theme 2: Social Opportunities

Children talked favorably about the social opportunities made available through breakfast clubs. They enjoyed spending informal time with peers in breakfast club before the start of the school day:

My favorite part is just like sitting down on the chairs and looking at each other and talking to each other (Girl, aged 10 years)

Breakfast clubs allowed children to make new friends:

You can meet all your friends and everything and you can meet new friends (Boy, aged 9 years)

Moreover, children were provided with opportunities to socialize with peers that they would not otherwise spend time with:

If you make friends with Key Stage Two boys they have to go over the road so you- you can’t see them at play, only at breakfast club (Girl, aged 7 years)

In one breakfast club that catered to children from two different schools, children had formed friendships with peers that they typically would not have encountered outside of breakfast club:

Me and [child] have friends that are from here and [other school] (Girl, aged 10 years)

Children’s views suggested that breakfast clubs provide a valuable and unique social outlet before the start of the school day. In particular, children enjoyed having informal social time prior to class time and valued being able to make new friends.

#### Theme 3: Positive Start to the School Day

Supporting the views of parents, children suggested that they enjoyed the time that they spent in breakfast club:

I love it there ’cause it’s so much fun (Boy, aged 4 years)

None of the children suggested that they disliked attending breakfast club and in some cases breakfast club was viewed as a better option than others available to children before the start of the formal school day:

If you don’t go to breakfast club you’re like a bit bored and when you go into school you’re like you still have loads of energy inside you and you’re just like messing about (Girl, aged 11 years)

Children also believed that breakfast club helped to prepare them for the school day by making them feel more alert:

At breakfast club like you have fun and you do some stuff energetic and it gets you like awake for when you come into school and you’re not just dosing off in your chair and that (Girl, aged 11 years)

It was further suggested that breakfast club attendance prevents children from being late:

It gets me to school earlier so I don’t miss half my lessons on maths (Boy, aged 7 years)

However, some children implied that they were tired in the mornings before breakfast club:

Facilitator: How do you feel in the morning before you’ve got to come into breakfast club?

Child 1: Tired (Girl, aged 10 years)

Child 2: Exhausted (Boy, aged 10 years)

Though it should be noted that it was not possible to ascertain whether children would have been just as tired getting up to attend school at normal time. Also, children felt fine once they were at breakfast club and as mentioned previously, none of the children proposed that they disliked attending breakfast club:

I cheer up when I actually get there and it’s alright (Girl, aged 11 years)

#### Theme 4: School Practicalities

It was evident through discussions with children that breakfast clubs generally offered a diverse array of enjoyable activities for children to partake in:

My favorite part is playing doctors “cause there’s my doctors case what I was playing with today (Girl, aged 5 years)

However, when asked about potential areas for improvement, children suggested that some of the equipment could be updated:

Child 1: Maybe more toys (Girl, aged 9 years)

Child 2: More? (Boy, aged 8 years)

Child 1: Well not more but all the ones that we’ve got are quite old and some of them are broken a bit (Girl, aged 9 years)

Moreover, children also wanted more of their peers to be able to attend breakfast club:

I would like more people to come in so you can make more friends (Girl, aged 7 years)

## Discussion

The current study aimed to investigate the advantages and disadvantages of school breakfast clubs according to parents, children and school staff. Findings showed that breakfast clubs have multiple positive factors that have the potential to impact social, behavioral, and educational outcomes for children. The opportunity to consume breakfast at school was viewed favorably by parents, staff, and children because it meant that children who skipped breakfast at home had an additional opportunity to access a breakfast meal before the start of the school day. This supports arguments posed by policymakers (26) and charities (27) to suggest that by ensuring children have access to a breakfast meal breakfast clubs could potentially reduce child hunger and poverty. Additionally, breakfast clubs might influence children’s breakfast habits and food preferences as some children were more likely to consume breakfast and try new foods at breakfast clubs than they were at home. Given the potential for breakfast clubs to have a substantial impact on children’s breakfast habits and preferences, it is essential that foods and drinks served within breakfast clubs are closely monitored to ensure they are nutritionally sound and fully compliant with school food standards. This is a key as the current findings showed that despite schools having access to guidance on foods that should be served in breakfast clubs, food-based standards are not being fully implemented meaning that children are able to consume foods such as chocolate spread for breakfast. Similar concerns regarding a lack of adherence to school lunch standards were raised within the SFP (10). These findings have important implications for future planning and implementation of school food as they suggest that further investigation is needed to determine why schools do not follow available food-based standards and how these standards can be improved to make them more accessible to those responsible for school food.

As well as consuming breakfast in breakfast clubs, children in the current study were able to partake in a multitude of semistructured play activities with peers and staff. The social opportunities afforded to children through breakfast clubs were thought to be unique, especially because they allowed children to spend time with peers that they might not otherwise have the opportunity to spend time with. Through play and shared activities, children learn an array of skills necessary for successful social interactions, including cooperation and conflict resolution (28). It is, therefore, plausible to argue that it is essential to encourage children to play together in order to allow them to develop favorable social abilities. However, the number of play opportunities available to children in schools has decreased dramatically in recent years, particularly because break times have been markedly reduced in favor of a greater emphasis on academic activities (29, 30). More recently, parental concerns over children’s safety have also been highlighted as a factor responsible for the reduction in the number of play opportunities available to children (31). As breakfast clubs offer children a safe environment in which to engage with their peers, it is possible that breakfast club attendance could increase children’s opportunities for social interaction and in turn impact upon the development of their social abilities and relationships with others.

Further advantages of breakfast clubs described in the current study pertained to broader impacts such as the calming effect that breakfast clubs were thought to have on children and their family routines in the morning, and the support that breakfast clubs offered to working parents. For mothers, the availability of childcare is a critical factor in their decision to go out to work (32) and with an increasing retirement age in the UK, parents of school-aged children are less able to rely on their extended family for help with childcare so accessible childcare provision needs to be in place to support families in pursuing and sustaining employment (33). Moreover, in the policy document “More Affordable Childcare” (34), the utilization of school premises has been proposed as a means of increasing available childcare particularly outside of formal school hours thus in light of the current findings it could be argued that investment in breakfast clubs should be recognized as support for an intervention that helps families beyond the provision of a breakfast meal.

However, a number of disadvantages of breakfast clubs also became apparent through discussions with parents, staff, and children. As well as concerns discussed previously regarding some of the foods served in breakfast clubs, further concerns were raised surrounding practical issues such as problems with staff being taken from other school duties to run breakfast clubs and the potential for children to be excluded from attending breakfast club due to the cost of attending. Additionally, there was some apprehension among staff that children attending breakfast clubs spend an extended amount of time in school, which might have a negative impact on their family relationships. These disadvantages illustrate that while breakfast clubs are generally well received and possess a number of beneficial features that have significant implications for children, families, and schools, there are practical aspects of breakfast clubs that need to be addressed to ensure that they continue to meet the needs of the children who attend as well as their parents and school staff.

Overall, the current findings offer a unique and timely contribution to the existing research literature on breakfast clubs in the UK by presenting the views of parents, school staff, and children within a single study. While the findings of the current study provide a useful starting point for further investigation into school breakfast clubs, it is important to highlight that data were collected from a purposive sample limited to a small number of schools in one area of the UK so it cannot be assumed that these findings would generalize to other areas of the UK. Future studies would benefit from extending the scope of the current investigation to include multiple areas spanning different levels of deprivation to see whether the same advantages and disadvantages are identified in different areas. It would also be useful to actively target fathers to be interviewed as they might offer a different parental perspective to that presented by mothers in the current study.

Despite some minor limitations, the present evidence provides a useful insight into the advantages and disadvantages of breakfast clubs within low-income areas of the UK. The findings hold particular relevance in the context of the SFP as breakfast clubs are currently being implemented in England’s “poorest schools” (10, p. 188) where breakfast provision is unavailable. The findings of the current study could therefore be utilized by policymakers to inform implementation and development of these new breakfast clubs.

## Conflict of Interest Statement

The authors declare that the research was conducted in the absence of any commercial or financial relationships that could be construed as a potential conflict of interest.
